# Shared decision-making in the context of COVID-19

**DOI:** 10.5935/0103-507X.20200034

**Published:** 2020

**Authors:** Glauco Adrieno Westphal, Juliano Ramos

**Affiliations:** 1 Unidade de Terapia Intensiva, Centro Hospitalar Unimed Joinville - Joinville (SC), Brasil.

Given the pandemic caused by severe acute respiratory syndrome coronavirus 2 (SARS-CoV-2), multiple treatments have been proposed for coronavirus disease 2019 (COVID-19), although there is no evidence yet that supports the use of any therapeutic option specific to the disease. Different entities, such as the National Institutes of Health (NIH), the Surviving Sepsis Campaign (SSC), the *Sociedade Brasileira de Infectologia* (SBI) and the *Associação de Medicina Intensiva Brasileira* (AMIB), do not recommend the use of specific therapies for COVID-19 (e.g., hydroxychloroquine, azithromycin, lopinavir/ritonavir, tocilizumab, immunoglobulin, etc.) until there is consistent evidence to support them in terms of both their efficacy and safety.^(1-4)^

On the other hand, suggestions regarding the use of chloroquine or hydroxychloroquine in severe cases of COVID-19 and the popularization of the topic generated certain expectations from the lay community, which requests and sometimes demands prescriptions for these drugs. Despite opinion 04/2020 of the Federal Council of Medicine (*Conselho Federal de Medicina* - CFM) reiterating that there is no solid evidence of the effect of these drugs on the prevention and treatment of COVID-19, the CFM considers their prescription possible, provided that prescriptions are made within a shared decision-making process in which the doctor explains to the patient and/or family members the weakness of the current evidence as well as the risks and benefits involved in the treatment.^(5,6)^

## Shared decision-making

Shared decision-making finds support in the ethical principle of beneficence and nonmaleficence. It aims to involve patients and/or family members in decisions related to clinical care and should be part of clinical practice. Shared decision-making means respecting the autonomy of patients and ensuring care that is consistent with their values and preferences. Therefore, the participation of patients and/or family members in decision-making should be considered when there are uncertainties about the benefits or a possibility of risks associated with any intervention. Generally, the understanding of patients and/or families when there is a decision to be made is achieved by discussing the pros and cons of existing options.^(7-10)^

## Practical suggestions for the shared decision-making process

### Define priority issues

Shared decision-making should be reserved for and should be used proactively in situations of uncertainty about risks and benefits or when decisions involve individual preferences and values, which are sovereign. Possibilities should be presented as options, and risks and benefits should be clarified, but the options should not presented as recommendations or impositions.^(7,9,10)^

### The interpersonal relationship should be healthy

A healthy interpersonal relationship during the decision-making process is based on helpfulness and must be egalitarian, empathetic and respectful, which means it must be exempt from value judgments about decisions. The physician’s acceptance of the patient’s and/or family’s decisions should be unconditional. A lack of acceptance weakens trust - a key element of an healthy interpersonal relationship.^(7)^

### Structure the communication

A structured conversation is the best way to convey complex information and help with the decision-making process. We suggest following the 12 basic rules of adequate communication used in the OPTION protocol (Table 1), a tool for measuring the quality of communication to guide the shared decision-making process with the patient and/or family. As evidence, the content of the conversation and its steps should be recorded in the medical records.^(11,12)^

**Table 1:** Essential aspects of the family conference for shared decision-making and suggestions for documentation in the medical records

	Essential aspects of shared decision-making	Suggestions for documentation of the family conference for shared decision-making in the medical records
1a	Identify the participants	We met today at ___/___ hours with Mr(s). ________________________ ( ) and/or his/her relatives ( ) for clarification of his/her clinical condition and the definition of joint decisions between patients and/or family members and the health care team regarding the actions to be taken. The family members ___________________________ and the following member(s) of the health care team _________________________________________________ were present at the meeting.
1b	Identify the problem that requires shared decision-making	The problem brought to the attention of the patient and/or family members for shared decision-making was ______________.
2	Explain that there is more than one way of dealing with the identified problem	It was explained to those present that there is more than one way to address the situation and that ...
3	Give options, which may include the option of "no action"	... the existing options for the case were listed and are as follows: _____________________. It was clarified that "no action/decision" is also an option ______.
4	Explain the pros and cons of each option	The pros and cons of each of the options were clarified, notably: _____.
5	Ask about the expectations (or ideas) of the patient about how problems should be managed	When asked, it was observed that the expectations of the patient and/or family members regarding the exposed problem are __________________________________________________________.
6	Ask about the concerns (fears) of patients about how problems should be managed	And that his/her fears and uncertainties about the case are _____________________________________. The uncertainties cited were discussed and clarified.
7	Check whether the patient understood the information	After asking the patient and/or family members about what they understood so far, it was observed that there was a *good*/*bad* understanding of the situation and ...
8	Offer explicit opportunities to ask questions	... they were given the opportunity to ask questions, which were answered.
9	Ask about the patient's preferred level of involvement in shared decision-making	When asked to what degree they would like to participate in the decisions, the patient and/or family said that________________________________________________________.
10	Indicate the possibility of postponing decision-making	It was also made clear that the decision can be postponed and that it can be discussed between the patient and/or family members. The patient and/or family members preferred ____________________________________.
11	Indicate the possibility of reviewing the decision	We clarified that, if they want to review the decision at any time, this can be done.
12	Evaluate the preferred way for the patient to receive information to assist in the decision-making process (e.g., discussions, printed material, graphs, video or other media)	Finally, after being asked, the patient and/or family members said that the preferred way to receive information was through ( ) meetings, ( ) printed material, ( ) graphs, ( ) video, ( ) other: ___________.

### Be cautious when making any recommendations

Contrary to common sense among health professionals, some communication models for shared decision-making suggest not generating direct recommendations in order to avoid imposing the values of the professional on the decisions of others. On the other hand, many patients and/or family members ask for a doctor’s recommendation, and the lack of an opinion can amplify emotional stress. In these cases, the professional’s opinion should revisit the risks and benefits, indicating the possibility of postponing the decision or even reviewing it in the future. Providing emotional support during that conversation may be necessary.^(7)^
